# Evaluating a Longitudinal Cohort of Clinics Engaging in the Family Planning Elevated Contraceptive Access Program: Study Protocol for a Comparative Interrupted Time Series Analysis

**DOI:** 10.2196/18308

**Published:** 2020-10-16

**Authors:** Rebecca Grace Simmons, Kyl Myers, Alexandra Gero, Jessica N Sanders, Caitlin Quade, Madeline Mullholand, David K Turok

**Affiliations:** 1 Division of Family Planning Department of Obstetrics & Gynecology University of Utah Salt Lake City, UT United States

**Keywords:** contraception, family planning, contraceptive initiatives, study protocol, reproduction, contraceptive, reproductive health

## Abstract

**Background:**

Access to high-quality, comprehensive contraceptive care is an inherent component of reproductive human rights. However, hindrances to specific aspects of contraceptive provision, including availability, accessibility, acceptability, and quality, continue to perpetuate unmet needs. The state of Utah has recently passed a series of contraceptive policies intended to improve contraceptive access. Despite these positive changes to theoretical access, fiscal appropriations to support the implementation of these policies have been minimal, and many individuals still struggle to access contraception.

**Objective:**

The Family Planning Elevated Contraceptive Access Program (FPE CAP), part of a larger statewide contraceptive initiative, specifically aims to improve contraceptive access within health clinics. This paper describes the study protocol for evaluating the success of FPE CAP.

**Methods:**

Health clinics apply for membership in the FPE CAP. On acceptance in the program, they receive a cash grant for clinical supplies, equipment, and personnel expenses; reimbursement for contraceptive services and methods for eligible clients; technical support, training, and proctoring on counseling and providing all methods of contraception; method stocking of intrauterine devices and implants; and demand generation activities, including local media campaigns, to inform community members about the FPE CAP and possible eligibility. FPE collects monthly service delivery reports from participating clinics for evaluation purposes. The primary outcomes of FPE CAP are level and trend changes in contraceptive service delivery among individuals earning ≤138% federal poverty level (FPL) following membership in FPE CAP and among FPE CAP clients earning between 139% and 250% FPL (including those ineligible for Medicaid) compared with historical data and control clinics. To assess this, we will conduct comparative interrupted time series analyses assessing the level and trend changes in intervention and control clinics 12 months before the intervention, for the 2-year duration of the intervention, and for the subsequent 12 months following the intervention.

**Results:**

We found that the study is adequately powered (>80% power) with our planned number of clinics and the number of months of data available in the study. To date, we have successfully completed the recruitment and enrollment of 8 of the expected 9 health organizations and 4 of the control clinics. Completed health organization enrollment for both intervention and control organizations is expected to be completed in December 2020.

**Conclusions:**

The study aims to provide insight into a new approach to contraceptive initiatives by addressing comprehensive aspects of contraceptive care at the health system level. Ongoing state policy changes and implementation components may affect the evaluation outcomes.

**International Registered Report Identifier (IRRID):**

DERR1-10.2196/18308

## Introduction

### Background

Contraception has been a primary tool to achieve reproductive justice, allowing people to plan their families as they see fit [1]. Acceptability of family planning as a human right has increased and so have policies and approaches to expanding comprehensive access to a wide range of contraceptive methods. In the United States, there have been a number of proactive family planning–friendly policies, including mandated contraceptive coverage by insurers, telehealth contraceptive counseling, web-based provision of short-acting hormonal methods such as oral contraception, over-the-counter availability of emergency contraception, and pharmacy dispensing authorization of hormonal contraception without prescriptions [2-4].

Despite these advances, obstacles to comprehensive contraceptive access remain. An estimated 38 million women in the United States have unmet need for contraception, 20 million of whom are in need of publicly funded family planning services [5]. Furthermore, the majority of studies of unmet need focus on women rather than the more appropriate inclusion of any person who can become pregnant and any person that can contribute to a pregnancy. Inclusion of the contraceptive needs of men and nonbinary individuals likely increases the estimated number of people who need contraception. Meeting the needs of all people of reproductive age also highlights the importance of progress toward comprehensive contraceptive care that incorporates the needs of sexually expansive and gender-expansive individuals.

Contraceptive provision that supports reproductive autonomy, as outlined in the tenets of reproductive justice, involves several components that must work in tandem to reduce unmet need [1]. Several theoretical frameworks, including the Availability, Accessibility, Acceptability and Quality framework, the access framework, the 5As of access by Penchansky and Thomas [6], the Bruce-Jain Quality of Care framework, and the Human Rights–Based Family Planning Framework, are in agreement on many of the various attributes required for comprehensive, equitable contraceptive access, including availability, accessibility, acceptability, and quality (Textbox 1) [6-10]. Barriers to these components can be identified across policy and service delivery levels and within individual experiences [11-13].

Barriers to each of these aspects of contraceptive provision exist in the state of Utah and throughout the United States. Although some aspects of contraceptive provision in Utah do have similarities to other states, the state has a unique combination of geographic and political aspects that may add complexities to an individual’s contraceptive access.

**Aspects of successful family planning provision included in multiple frameworks (Availability, Accessibility, Acceptability and Quality framework, Penchansky and Thomas J, Human Rights–Based Family Planning Framework, and Bruce-Jain Quality of Care).**

Availability:

- Is the method offered to individuals through standard channels of care when they want it?

Accessibility:

- Can an individual get the method they desire?
- This can encompass financial, logistic, or geographic barriers

Acceptability:

- Is the method marketed or offered or distributed in a way that aligns with medical ethics, cultural, and individual values?

Quality:

- Is the method provided in a way that meets the highest medical and ethical standards?
- This includes ensuring dignity and respect for clients, informed consent through provision of accurate scientific information, ensuring client privacy and confidentiality, and meeting technical competence in provision of methods

### Availability Barriers

Despite widespread method availability in the United States and Utah, barriers still exist. These availability barriers manifest primarily within the service delivery sector, through factors such as a lack of skilled providers, a limited selection of methods at clinics, and stockouts [14,15]. Subsidized or discounted purchasing, in the form of 340B federal drug pricing or group purchasing, has the potential to reduce the upfront cost of stocking methods (Multimedia Appendix 1 provides a glossary of health policy terminology). However, not all clinics providing contraception to low-income clients meet the 340B criteria, and discounted prices through group purchasing models may still be prohibitively high for smaller clinics. This leads clinics to make fiscal decisions about which methods to purchase and subsequently impacts the availability of methods. In addition, varying demand for the most expensive methods, such as intrauterine devices (IUDs) and contraceptive implants, increases the likelihood of stockouts, particularly among smaller clinics [16].

Furthermore, the availability of comprehensive contraceptive offerings including both long-acting reversible contraceptives (LARCs) and lesser used methods, such as fertility awareness–based methods, is limited in most publicly funded health clinics [17]. Expanded method choice is associated with increased utilization and method satisfaction among users [18], making a comprehensive contraceptive offering an important component of contraceptive availability.

### Accessibility Barriers

The cost of health care, including contraceptive services, is a known barrier to use, particularly for individuals with low incomes. Several studies have demonstrated the removal of cost barriers, leading to increased utilization of methods, including LARC methods, which have the highest upfront costs [17,18]. Until recently, Utah was 1 of only 7 states that had not either expanded Medicaid through the Affordable Care Act or adopted a Medicaid Family Planning 1115 Waiver (Multimedia Appendix 1). As a result, an estimated 215,000 women in the contraceptive coverage gap needed publicly funded family planning services [19]. In addition, approximately 20% of existing insurance policies in Utah are considered noncompliant with the Affordable Care Act and do not necessarily require insurers to cover contraceptive services and methods [20].

Geographic access barriers also exist in the state. Utah is one of the largest states in the United States, but one of the least populous states. Approximately 25% of the state’s population lives in rural or frontier areas, requiring traveling longer distances to receive care. Utah has a lower ratio of physicians per civilian population than the national average; this gap is particularly acute in rural health districts [21]. In 3 Utah counties, there are no primary care physicians [21]. This often translates into Utahns needing to factor transportation and time costs into their contraceptive utilization.

Recent changes in national Title X requirements resulted in Planned Parenthood withdrawing from Title X funding in 2019. Planned Parenthood clinics were Utah’s only Title X provider, and their withdrawal from the program’s federal funding has left Utahns without a single Title X provider. This has increased accessibility barriers to contraception for many primarily low-income and adolescent individuals.

### Acceptability Barriers

A major aspect of contraceptive acceptability in Utah is tied to the high rates of residents’ membership in the Church of Jesus Christ of Latter-Day Saints religion. Overall, 62% of people living in Utah report affiliation with this religion, although there are substantial regional differences across the state [22]. An even higher proportion (88%) of individuals serving in the Utah legislature identify as being affiliated with this religion [23]. Thus, much of the cultural acceptability of and policies related to contraception have roots in the dominant religion’s beliefs about sex and sexuality. This includes topics such as extramarital sex (and by extension, adolescent sex), nonheterosexual sex, and abortion [24,25].

As of 2019, state code permits sex education curricula in Utah schools to describe contraceptive methods. Before 2019, sex education curricula were not allowed to describe contraceptive methods. A yet-unpublished survey of sexual health knowledge among students at the University of Utah found that although 82% of respondents reported being sexually active, 52% reported only having received abstinence-based sex education, and 63% reported that they wished they knew more about specific contraceptive methods [26]. Thus, contraceptive knowledge among Utahns is low, and methods are subject to common myths and misconceptions. In one such example of these misconceptions, a Utah-based medical practice received media attention in 2019 for the *premarital exams*, which are essentially provider-based sexual education visits, primarily directed at women [27].

Contraceptive acceptability also pertains to providers, particularly regarding the acceptability of certain methods in specific circumstances or for particular individuals. For example, a presurvey provided at a Utah contraceptive training conference found that 65% of respondents (n=29) perceived that intrauterine placements were less uncomfortable for recipients if provided during menstruation [28]. In addition, research on sexual minority women in Utah found that these individuals are less likely to report having had a provider offer contraception or discuss pregnancy intentions or reproductive life planning [29,30].

### Quality Barriers

Attributes of quality contraceptive provision are evolving. For example, new evidence supports patient-centered decision making, which places the client as the primary decision maker in a contraceptive visit, with the provider in a supportive ancillary role [31]. Recommendations for high-quality contraceptive visits now include providing anticipatory guidance for managing side effects, advancing the provision of emergency contraception to individuals who would like it, and identifying alternative or backup methods for individuals who are unhappy or dissatisfied with their current method [31]. Yet, updated contraceptive education is limited for many established providers in Utah, particularly individuals in primary care settings in rural and frontier areas who may find it difficult to stay current on sexual and reproductive health topics, given competing priorities and training.

### Current Policy Environment

In 2018, Utah legislators approved the request for a Medicaid 1115 waiver, expanding contraceptive coverage and family planning services for individuals with household incomes below 100% federal poverty level (FPL). In 2019, this waiver was absorbed into a larger targeted Medicaid expansion during the 2019 legislative session. The family planning waiver estimated increased contraceptive coverage to approximately 11,200 low-income individuals in the state [32]. The broader targeted expansion estimated that between 70,000 and 90,000 people would have increased coverage, which would include family planning services [33]. Finally, in January 2020, Medicaid was further expanded in the state to meet federal eligibility guidelines for individuals at or below 138% FPL.

However, theoretical access through coverage-related policy change does not always translate to actual access. Opportunities for successful policy implementation exist at both the individual and health systems level and include educating Utahns about the new coverage options, enrolling eligible individuals, assisting newly enrolled individuals in using health care services, training providers on comprehensive method counseling and provision, purchasing necessary devices and equipment for care, and addressing increased demand within the clinic workflow. These essential aspects were not included in the fiscal appropriations of the expansion bill in 2019, nor in the expansion of 2020. In addition, the expansion of coverage to 138% FPL does not address contraceptive coverage for individuals who are documented immigrants (eg, have visas or green cards) or who are undocumented and ineligible for Medicaid. Furthermore, many individuals who are eligible for coverage through the Affordable Care Act marketplace struggle to pay for health care, have high deductible plans, or no prescription coverage. Research has demonstrated that the need for subsidized contraceptive care continues to be critical for individuals up to 250% FPL [33].

Family Planning Elevated (FPE) is a statewide contraceptive initiative that was developed in part to support the implementation of the evolving Medicaid policy and provide evidence about additional state-specific opportunities to further improve contraceptive availability, acceptability, accessibility, and quality in Utah. This paper provides an overview of the FPE Contraceptive Access Program (FPE CAP)—a subset of the initiative specifically aimed at (1) strengthening clinic capacity for expanded contraceptive service delivery to individuals covered by the new Medicaid expansion (>138% FPL) and (2) providing comprehensive no-cost contraceptive care to individuals between 139% and 250% FPL.

## Methods

### Study Design

The primary purpose of this quasiexperimental study is to determine whether the FPE CAP increased clinic provision of contraceptive services to Medicaid-insured individuals at 0% to 138% FPL and uninsured and underinsured individuals between 139% and 50% FPL, compared with clinics that did not receive the intervention program. A total of 9 health organizations will be selected in 3 cohorts, spaced 6 months apart, for a 2-year membership in the FPE CAP (February 2019 to February 2022) and will be matched in a 1:1 ratio to control clinics within the state to compare contraceptive service outcomes. The University of Utah Institutional Review Board (#00117213) approved this study.

Participants in this study are health organizations that are selected for membership within the FPE CAP. To be eligible for FPE CAP membership, a clinic must (1) serve a patient population that is at least 25% uninsured or underinsured; (2) accept Medicaid; (3) primarily serve clients below 250% FPL; and (4) be enrolled in, or eligible for, the federal 340B drug pricing program. Selection priorities are given to health centers in rural areas of Utah and those in counties with high unintended birth rates and low health care coverage rates.

The criteria for matching control clinics in Utah include clinic size (volume, estimated by the number of providers), a similar proportion of uninsured and underinsured population served, geographic denotation (urban, rural, and frontier), and acceptance of Medicaid. Eligible controls are those that both meet matching criteria and confirm that they do not intend to apply for the FPE CAP but are willing to provide service delivery data to the program as a control clinic. On selection of intervention clinics, control clinics were identified and recruited. Control clinics provide nominal compensation (US $100 per month) for the provision of their data over the study period.

Clinics interested in participating in the FPE CAP apply for membership in 1 of the 3 cohorts. In their applications, clinics provide FPE a self-assessment of the availability and accessibility of contraceptive methods in their individual organizations (eg, whether they had certain methods available, whether providers were trained on IUDs and implants, etc). On selection, receive a multifaceted intervention that includes several specific components (Table 1).

**Table 1:** Overview of Family Planning Elevated Contraceptive Access Program activities and their alignment with theoretical aspects of family planning care.

FPE CAP^a^ intervention activities	Theoretical aspects of contraceptive provision addressed
FPE CAP membership specifically targeting geographic areas with high contraceptive need and low comprehensive contraceptive provision	Increased geographic accessibility of comprehensive contraception in rural and frontier areas
Cash grant to support clinic capacity to provide contraception (ie, staffing, procurement of instruments and supplies)	Improved availability of comprehensive contraception at clinics
No-cost contraception for all reversible methods of contraception for both eligible individuals between 139% and 250% FPL and individuals between 0% and 250% FPL who are Medicaid ineligible_b_	Financial accessibility of comprehensive contraceptive methods
Provider and clinic training on contraceptive provision and contraceptive counseling (eg. Contraceptive Education and Training conference, in-clinic training, 1:1 proctoring)	Increased availability of comprehensive methods, including provider-dependent methods (eg, long-acting reversible contraceptives, vasectomy) Increased quality of family planning counseling and method provision in clinics
Tailored technical offerings to clinics, specific to requested areas of need (eg, adolescent friendly services, proctoring on intrauterine devices and implants)	Increased quality of family planning counseling and provision in clinics
Tailored contraceptive social media campaigns specific to different communities, directing individuals to participating FPE CAP clinics	Improved acceptability of comprehensive contraceptive methods within specific communities

^a^FPE CAP: Family Planning Elevated Contraceptive Access Program.

^b^FPL: federal poverty level.

First, each FPE CAP member receives a cash grant up to US $100,000 based on their budgeted need for equipment, supplies, or supplemental staffing to expand service capacity. Provision of provider-dependent methods such as IUDs and implants requires specific equipment for insertion and removal, and many clinics struggle with the initial cost of purchasing instruments, examination tables, and sterilization equipment. The expected increase in demand for health care services stemming from Medicaid expansion may place additional burdens on already busy clinics, and covering the initial costs of offering family planning services can increase immediate clinic capacity.

Member clinics must be able to provide all reversible methods of contraception to individuals between 139% and 250% FPL (and Medicaid-ineligible individuals between 0% and 250% FPL) at no cost to clients for the duration of the 2-year program, and clients are able to switch methods or discontinue at any time. To make this feasible, FPE CAP provides member clinics with a continuous supply of long-acting methods, including IUDs and implants, as these often have high upfront procurement costs, even with 340B pricing or other purchasing subsidization. In addition, FPE CAP reimburses clinics for all reversible methods and contraceptive services (including counseling, procedures, etc) at Medicaid rates. Clinics capable of providing vasectomy receive Medicaid reimbursement for that service as well. These reimbursements are meant to mimic the experience of a Medicaid family planning waiver expansion to 250% FPL or similar coverage expansion that could be implemented at a policy level and demonstrate the existing demand among this population. The FPE CAP tracks the supply and provision of all methods using both monthly service delivery data and monitoring data collected qualitatively in the quarterly report calls with individual clinics.

The full patient care team from administrators to providers at member clinics receives tailored education and training on a variety of topics, including person-centered contraceptive counseling, IUD and implant placement and removal, barriers, fertility awareness–based methods, clinic workflow, billing, coding, and other areas of need jointly identified by each clinic and the program team. Clinic staff at all levels, including front-desk staff and medical assistants, will be involved in education and training to support the systems’ capacity for contraceptive provision throughout the entire clinic. FPE CAP members are asked to identify clinic champions at the provider, medical assistant, and administrative levels to support the project and to increase the likelihood of sustainability after the program ends. Clinic providers who receive IUD and implant training will also receive onsite proctoring and mentorship, clinical assistance with complex cases, and additional specific training, such as immediate postpartum insertions. Providers also have access to an on-call nurse practitioner who specializes in family planning care to support and troubleshoot any issues.

Finally, FPE CAP will develop demand generation activities and community outreach tailored for each member clinic to reduce unmet need for contraceptive services within the clinic communities. These activities will occur after the FPE CAP service intervention is successfully implemented within each clinic to ensure that the clinic has the capacity to increase service delivery while maintaining service quality.

### Data Collection

To assess the primary outcomes, both intervention and control clinics agree to provide monthly service delivery data from their electronic health record systems to use for the program evaluation, following a standardized form developed by FPE (Multimedia Appendix 2). Clinics agree to provide data on (1) total clinic volume (men, women, and children); (2) all clinical services (including noncontraceptive services) provided to women aged 18 to 50 years each month (including International Classification of Diseases, 10th Revision, codes, Evaluation and Management codes, Healthcare Common Procedure Coding System, and Current Procedural Terminology codes); and (3) sociodemographic variables of interest for each visit, including age, race, ethnicity, insurance status (private insurance, Medicaid, FPE CAP, self-pay, and other), and provider type. All clinics provide these data beginning from January 2018 to 12 months after their implementation period ends.

Other components of the FPE CAP intervention, including education, training, and demand generation, will be captured with subevaluations. These secondary analyses will assess changes in both knowledge and self-efficacy among providers, a media campaign evaluation, and a detailed process evaluation. Clinic service delivery quality will be assessed through yearly clinic visits by FPE staff and through periodic implementation of client exit surveys of individuals who received contraceptive services at member clinics. Additional subanalyses, including comparisons between FPE CAP clinics, are also planned.

FPE also includes monitoring and process evaluation components for the project, which assess aspects of adherence to the program and track components of dose and frequency as they relate to programmatic success. FPE’s process evaluation uses the Consolidated Framework for Implementation Research as the formal tool for assessment. Further information about these components of the project has been reported elsewhere [34]. Our publication on the Open Science Framework provides an overview of all FPE monitoring and evaluation activities [35].

### Statistical Analyses

Primary outcomes of the program will be assessed using comparative (intervention vs control), multiperiod (before, during, and after), interrupted time series analyses (ITSAs) [36,37]. ITSAs have increasingly been demonstrated to be reliable assessments of community interventions and implementation research, providing rigorous outcome assessments in circumstances where randomized controlled trials are infeasible [38,39]. For our purposes, our primary outcomes are increase in total contraceptive services among individuals between 139% and 250% FPL or who are undocumented and increase in total contraceptive services among individuals eligible for Medicaid. Total contraceptive services refer to any contraceptive service (ie, counseling, method provision, method removal, etc) that the clinic provides.

The 2 general approaches to ITSA are autoregressive integrated moving-average models [40] and ordinary least squares (OLS) models designed to adjust for autocorrelation [41]. We will use an OLS model because it is often more flexible and broadly applicable in an interrupted time series context than autoregressive integrated moving-average models [42,43]. To adjust for autocorrelation, we will fit an OLS model with Newey-West standard errors, which assume the error structure to be heteroskedastic and possibly autocorrelated up to some lag [44]. After fitting our model, we will check if the number of lags included in the model to account for autocorrelation was correctly specified and adjust accordingly using the Cumby-Huizinga general test for autocorrelation [45].

We conducted a Monte Carlo simulation to assess the power to detect significant differences between intervention and control clinics. Using initial, preintervention data from 9 identified potential clinics, the 6-month average number of monthly contraceptive service provision was 3, 28.4, 16.8, 16.9, 13.8, 23.2, 21.9, 16.4, and 5.4, with a mean of 16.2 (SD 8.1) contraceptive services provided per month. We used this SD to provide the correct amount of between-clinic variation in the simulation, with the assumption that this same SD will apply across all 3 periods (before, during, and postintervention) and would be equal between the 2 groups. We assumed that the 2 groups would be equal in the preintervention period and would have similar regression lines with a slope (background trend) of a 0.25% increase in contraceptive service provision per month. We assumed that this preintervention trend would continue in the control group across the intervention and postintervention periods, without interruption, and would be represented by one regression line across the entire study period. In the intervention group, we assumed an immediate jump (interruption) of 12 (which is about a relative 75% increase because the clinics now would have no-cost contraception to offer eligible clients), followed by a greater slope, now 1% per month in the intervention period (as providers and clinics would show improvements in contraception counseling and method availability). In the postintervention period, where no-cost contraception would no longer be available to provide to the clients, we assumed an immediate drop of 12 (losing the immediate gain at the beginning of the intervention period).

Using a normal random number generator, we generated a random data set based on our assumptions and performed the ITSA. We ran the simulation 1000 times and saved the significance determinations (*P*<.05). The mean of significance determinations, which is identical to the proportion of samples with statistical significance, is identical to the statistical power. The findings from our simulation are presented in Table 2. The Stata code for this power calculation, including notations around our assumptions, is available on the Open Science Foundation website [35]. All analyses were conducted using Stata version 15 or higher.

**Table 2:** Monte Carlo simulation for Family Planning Elevated Contraceptive Access Program study power calculation.

Comparison	Estimate (absolute % difference)	Power (%)
Preintervention period group difference in trend (slope)	0	11^a^
Intervention period group difference in trend	0.75	>99
Postintervention period group difference in trend	−1.25	86
Intervention period group difference in interruption (immediate effect of introducing intervention)	12	88
Postintervention period group difference in interruption (immediate effect of stopping intervention)	−12	93

^a^No difference assumed, so low power is expected.

## Results

We found that the study was adequately powered (>80% power) with our planned number of clinics and the number of months of data available in the study. To date, we have successfully completed the recruitment and enrollment of 8 of the expected 9 health organizations and 4 of the expected 9 control clinics. Completion of health organization enrollment for both intervention and control organizations is expected finish in December 2020.

## Discussion

Expanding family planning access through policy change is a critical component of reducing unmet need for contraceptives. However, changes to legislation require implementation support to translate policy change into literal, on-the-ground access at service delivery, community, and individual levels. Our statewide FPE program to align service delivery capacity with contraceptive policy is novel but is grounded in evidence-based theory on existing barriers to contraceptive access.

Implementing such a program is not without challenges. First, the program incorporates a diverse network of clinics, including federally qualified health centers, private clinics, and city and county clinics. Incorporating activities into multiple practices with varied protocols, policies, and norms involves significantly tailoring the intervention to meet individual clinic needs. For example, participating FPE CAP members invoice the FPE CAP for contraceptive services provided to eligible individuals, yet clinics vary widely on billing practices and capacities. Thus, reimbursement requires a tailored approach to education, training, and follow-up of clinic administrative staff as well as programmatic flexibility to accept and correctly interpret program billing inputs provided variably.

In addition, clinic capacity varies widely among FPE CAP members. In some participating clinics, all providers are already trained to provide LARC methods, whereas in others, no or very few providers have received training. Developing training materials that identify priority foci that apply to the full care team is important, as it meets the individual training needs of clinics that require more assistance. For example, all clinics receive training on evidence-based practices for contraceptive counseling, reproductive justice, and implicit bias; however, for practical skills, such as LARC insertion and removal, clinics may receive more or less training and supportive supervision, depending on experience levels. As such, education and training approaches are essentially tiered, with basic education for all clinics and then specialized education and training for those clinics that require additional assistance.

Furthermore, FPE CAP is operating in a busy clinical environment within member clinics that provide a wide range of services in addition to contraceptive care. The program is among a number of competing priorities for these clinics, which provide high-volume care to low-income and marginalized communities. The program is attempting to strike a balance between reasonable intervention requirements and reducing the burden of implementation in clinics. This has had a decisive impact on many programmatic aspects, such as the amount and type of data collection required, the number of educational training required, the reporting requirements, and other components of the intervention. Cognizance of *initiative fatigue* as a component of intervention design is important to ensure program participation, completion, and compliance.

In addition, there are data collection challenges inherent to working across clinical electronic medical records (EMRs) and systems as well as incorporating needed variables into clinical practice. FPE CAP clinics and controls use various EMRs, each of which has a different capacity to pull reports and variables required for data collection. To address challenges in developing a standardized report that meets study guidelines, our evaluation team conducts in-person data meetings at the outset of membership to help the intervention and control clinics develop the report according to the program needs. There are also vast differences in how organizations and providers document and code clinical encounters. This will likely be a limitation in future analyses, despite FPE CAP efforts to improve clinical coding capacity by providing standardized training, visual aids, and other materials to incentivize improved coding practices.

Finally, FPE CAP operates within a continuously changing health policy landscape. The subsumption of the Medicaid family planning waiver into the larger Medicaid expansion was both a positive development for health care in Utah as well as a challenge to the initiative. Rollout of the policy was delayed while the new legislation was being finalized, requiring member clinics to adopt variable patient eligibility parameters at the start of the intervention, after the targeted Medicaid expansion was approved, and once again after the full expansion took effect. Additional changes may yet occur to state Medicaid, which is currently being implemented with controversial components that have not been previously approved by the federal government, including work reporting requirements and per capita caps. As decisions around these policy components are made and initially eligible individuals potentially lose coverage, there may be additional changes to FPE CAP operations, as the program strives to support individuals who fall in service gaps.

## Appendix

Multimedia Appendix 1 Health policy glossary.

Multimedia Appendix 2 Monthly service delivery report template.

## Abbreviations

EMR: electronic medical record
FPE: Family Planning Elevated
FPE CAP: Family Planning Elevated Contraceptive Access Program
FPL: federal poverty level
ITSA: interrupted time series analysis
IUD: intrauterine device
LARC: long-acting reversible contraceptives
OLS: ordinary least squares
