# Supplementary material for: Evaluating a Longitudinal Cohort of Clinics Engaging in the Family Planning Elevated Contraceptive Access Program: Study Protocol for a Comparative Interrupted Time Series Analysis
Source: JMIR Res Protoc. 2020 Oct 16;9(10):e18308. doi: 10.2196/18308 (PMC7600020; doi:10.2196/18308)
Supplement: Multimedia Appendix 1 [file resprot_v9i10e18308_app1.docx]

Appendix 1: Health policy glossary

**340B Drug Discount Program:** A US federal government program created in 1992 that requires drug manufacturers to provide outpatient drugs to eligible health care organizations and covered entities at significantly reduced prices.

**Federal poverty level:** The federal poverty level is a measurement developed to assess a person’s eligibility for different federal assistance programs. It changes every year and accounts for household size and income levels.

**Group purchasing:** This process allows clinics to aggregate their purchasing volume and use that leverage to negotiate drug and other healthcare discounts with manufacturers, distributors, and other vendors.

**Medicaid 1115 waiver:** These waivers expand coverage of specific Medicaid services (such as contraceptive services), while not providing to the full range of services covered under Medicaid.

**Title X:** This federal grant program provides annual monies to eligible clinics to ensure access to family planning and related preventive health services for low-income and uninsured individuals.
