# Supplementary figures and images for: Evaluating a Longitudinal Cohort of Clinics Engaging in the Family Planning Elevated Contraceptive Access Program: Study Protocol for a Comparative Interrupted Time Series Analysis
Source: JMIR Res Protoc. 2020 Oct 16;9(10):e18308. doi: 10.2196/18308 (PMC7600020; doi:10.2196/18308)

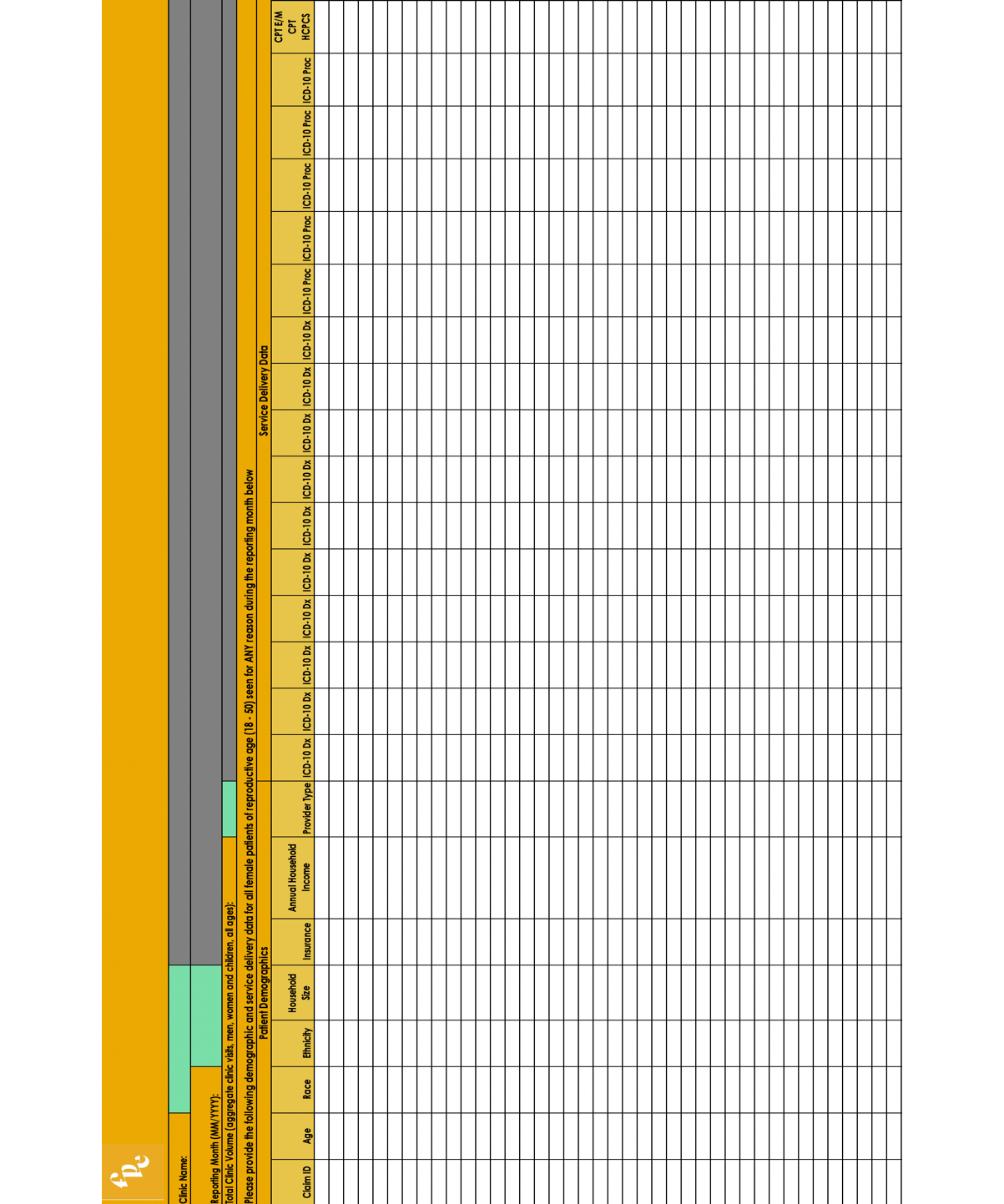

Supplement: Multimedia Appendix 2 [file resprot_v9i10e18308_app2.png]
